# Novel three-dimensional acellular dermal matrix for prepectoral breast reconstruction: First year in review with BRAXON^®^*Fast*

**DOI:** 10.3389/fsurg.2022.970053

**Published:** 2022-09-05

**Authors:** Giorgio Berna, Alessia De Grazia, Elisa Antoniazzi, Marco Romeo, Francesco Dell’Antonia, Stefano Lovero, Paolo Marchica, Christian Rizzetto, Paolo Burelli

**Affiliations:** ^1^ Plastic Surgery Department, Ospedale Ca’ Foncello, ULSS2 Marca Trevigiana, Treviso, Italy; ^2^ Breast Unit, Ospedale Ca’ Foncello, ULSS2 Marca Trevigiana, Treviso, Italy

**Keywords:** breast reconstruction, prepectoral, three-dimensional ADM, collagen matrix, safety

## Abstract

Implant-based breast reconstruction is part of breast cancer treatment, and increasingly optimized reconstructive procedures exploit highly biocompatible materials to ensure enhanced aesthetic-functional results. Acellular dermal matrices (ADMs) are collagen-based materials that made prepectoral implant placement possible, thanks to their bioactive antifibrosis action. Recently, the first three-dimensional ADM, BRAXON^®^*Fast*, has been produced. Its 3D design represents the technological evolution of BRAXON^®^ ADM, a flat collagen matrix, and allows for a time-saving complete wrapping of the synthetic prosthesis, thus creating a total biological interface on the implant with patient's tissues. Here, we report our experience on the first 23 eligible patients who received BRAXON^®^*Fast*-assisted prepectoral reconstruction. On a total of 27 breasts, the overall complication rate was 11.1%, including one minor seroma (3.7%), one case of necrosis (3.7%), and one implant removal due to infection. As new-generation devices, 3D ADMs showed an effective performance, allowing to reduce the overall exposure time for implant preparation and providing an optimal safety profile**.**

## Introduction

According to AIOM (Italian Association of Medical Oncology), about 55,500 breast cancers are diagnosed every year in Italy, and it is the most frequent form of cancer in the female population (1). Whenever applicable, breast reconstruction should be offered to patients undergoing mastectomy for either breast cancer treatment or as a risk-reducing procedure since it contributes to the preservation of patients’ quality of life (QoL), which is profoundly eroded by the whole oncologic patient status and the therapeutic process (2).

The last two decades have seen an increase in implant- and tissue expander-based immediate breast reconstructions, from 30% in 2007 to 54% in 2013 (3). Since the 1980s, submuscular techniques have been the standard practice for implant-based breast reconstruction (IBBR), and they are still widely used. However, problems of animation deformity and postoperative pain due to muscle detachment still raise concerns. Indeed, although contraindications related to patients' characteristics can shift the reconstructive plan to a submuscular approach, prepectoral breast reconstruction (PPBR) has gained popularity since it offers a more physiological reconstruction and obviates problems related to muscle detachment, and we expect it to become the gold-standard technique in IBBR (4).

PPBR consists in placing the implant above the pectoralis major muscle following nipple-sparing, skin-sparing, or skin-reducing mastectomies. Our senior author, together with the colleague Dr. Simon J. Cawthorn, first presented the novel one-step technique at the MBN Breast Meeting 2013 Congress in Milan: the positioning of a prepectoral breast implant covered by an acellular dermal matrix (ADM) in the place of the mammary gland. Since then, variations of the technique have also been described with the use of synthetic meshes, mainly a practice only supported by short-term economic reasons (5–8).

Nowadays, the most used devices in PPBR are biological membranes such as BRAXON® acellular dermal matrix (licensed by DECOMED, Marcon, Venezia, Italy). This device has been specifically designed for prepectoral IBBR, available as a preshaped, 0.6--mm-thin, unfenestrated porcine collagen membrane apt to be wrapped around the implant before implantation. Outcomes with this ADM have been widely described in the literature, even in the long-term and large cohorts, proving enhanced aesthetic and functional results, improved QoL, and cost-effectiveness compared to submuscular approaches (5, 9–16).

The breast implant wrapping ease allowed by the precut shape of BRAXON® ADM might have contributed to rendering it the most studied biological matrix for PPBR (17). In 2021, BRAXON® has been released in the new format BRAXON®*Fast* (DECOMED, Venezia, Italy). BRAXON®*Fast* is the first three-dimensional ADM indicated for PPBR. It shares with BRAXON® all the technical characteristics and biological features (origin and material, 0.6 mm thickness, no fenestration, bioactivity). Its novelty consists in the fact that it is not only preshaped but also already curved: one side appears like a dome to specifically follow the convexities typical of the anterior surface of a breast implant, while the second side is flat for covering the back of the implant. Such 3D conformation would speed up the implant wrapping process, limiting implant exposure and manipulation that could put sterility at stake. Here, we report on the safety and effectiveness of this new-generation 3D device, weighing up the practical implications of such a precurved matrix.

## Methods

Data of patients who underwent a nipple-sparing mastectomy and PPBR from January 2020 to December 2021 at “Ca' Foncello” Hospital (Treviso—Italia) were collected and evaluated. All surgeries were performed by two senior breast surgeons and two senior plastic surgeons. Mastectomies were either prophylactic (patients at high risk with a family history of breast cancer or with mutated susceptibility genes) or therapeutic (bilateral tumors). Both unilateral and bilateral breast reconstructions were performed, and all cases were BRAXON®*Fast*-assisted PPBR. In the case of unilateral reconstruction, when necessary, contralateral symmetrization was performed within the same surgery. Patients undergoing any fashion of breast reconstruction other than prepectoral were excluded from the current analysis, and only procedures with nipple–areola complex preservation were collected. Patients undergoing postoperative radiation treatment were excluded, as well as patients undergoing axillary lymphadenectomy. With a minimum follow-up of 3 months, we collected all complications, and their emergence rate was calculated on the breasts' total number. Complications rates were analyzed and compared to data currently available in the literature, including our previous experiences.

### Surgical technique with BRAXON^®^*Fast*

For this kind of reconstruction technique, patients were preoperatively selected for small to moderate ptosis, pinch test ≥1 cm on the upper pole, no history of radiotherapy to the breast, and no active smokers. Intraoperative selection after mastectomy was based on the presence of good viability of the mastectomy flap tested clinically (good refill, color, active bleeding from the edges).

All patients were administered antibiotic prophylaxis with Cefazolin 2 g before induction; if allergic to penicillin, Clindamycin 600 mg was administered. Preferred surgical access for nipple-sparing mastectomies was a radial italic-*S* incision, departing from the nipple–areola complex, or inframammary fold incision. Chlorhexidine skin antisepsis was always performed prior to incision and before reconstruction. Following mastectomy, both the prepectoral cavity and the prosthesis were irrigated with a saline and antibiotic solution (Clindamycin 600 mg) as part of the antiseptic procedure, and a suction drain was placed in the caudal part of the mastectomy cavity. After the procedure, all patients were checked for implant volume and breast symmetry with an implant sizer in a semisitting position on the operating bed.

**Figure 1:**
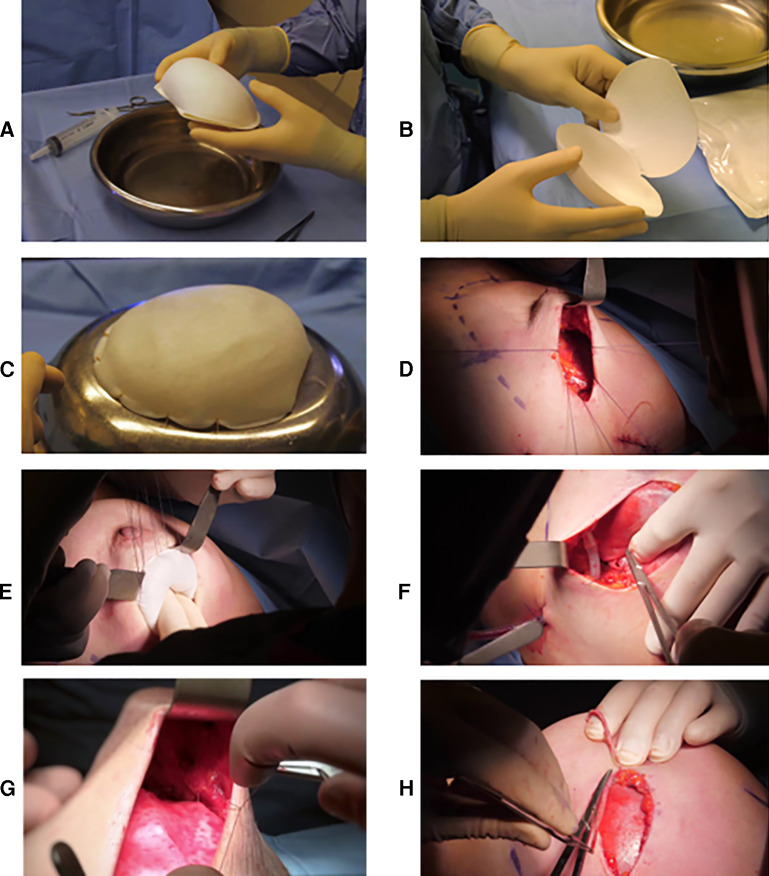
(**A**) BRAXON^®^*Fast* in its close and (**B**) open conformation before hydration. The precurved side and the flat side are visible. (**C**) Implant preparation. BRAXON^®^*Fast* completely covers the breast implant. Single stitches are used to close the matrix flaps. (**D**) Parachuting sutures used to facilitate implant positioning inside the breast pocket. (**E**) Implant positioned inside the breast pocket. (**F**) Fixation of the implant to the pectoralis major muscle fascia and (**E**) to the subcutaneous tissue. (**H**) Wound edges excision before closure.

BRAXON®*Fast* ADM is shown in Figures 1A,B. It was used to envelope anatomical implants (Mentor). After implant wrapping, the overlapping parts of the anterior and posterior ADM flaps are sutured together with 7–10 single stitches using Vicryl 3-0 absorbable sutures (Figure 1C). In the case of an excess of ADM, it was trimmed. We inserted the implant with parachuting sutures (Figures 1D,E) and stabilized the implant position by fixing BRAXON®*Fast* to the pectoralis major muscle fascia (Figure 1F) with three medial stitches, two at the inframammary fold and one in the superior part of the pocket. The matrix was stitched to the subcutaneous tissue to ensure mechanical stillness and intimate contact between the two (Figure 1G), and the pocket was closed laterally with at least two strata of stitches after excision of the wound edges (Figure 1H). All operators changed gloves before manipulating the ADM and the implant and before implanting the ADM-wrapped implant. If contralateral symmetrization procedures were programmed, mastopexy or reductive mammaplasty was performed simultaneously and tailored on the appearance of the reconstructed side.

## Results

A total of 23 patients underwent mastectomy and PPBR with BRAXON®*Fast* and suited the selection criteria, for a total of 27 breasts treated from January to December 2021. Relevant data are summarized in Table 1. Four patients underwent a bilateral reconstruction, while nine patients underwent contralateral symmetrization, accounting for a total of 13 bilateral procedures. The cohort had a mean age of 53.2 years, and the average length of hospital stay was 3 days. The average implant volume was 392 cc.

**Table 1:** Cohort data.

	Number (range)
Patients	23
Breasts	27
Unilateral procedures	10
Bilateral procedures	13
Symmetrization procedures	9
Bilateral reconstructions	4
Mean age (years)	53.2 (36–67)
BMI (kg/m^2^)	26.9 (23.6–34.1)
Smokers	0
Mean hospital stay (days)	3
Mean implant volume (cc)	392 (180–570)
Mean follow-up (months)	7.3 (3–11)

Complications rates were recorded and were categorized either as major complications, meaning those complications that required access to the operating room, or minor complications, indicating those that were resolved in outpatient conditions. The total complication rate was 11.1%, including two minor complications and one major complication.

Minor complications counted for one minor seroma, defined as a collection of fluid of 30–50 cc solved with a maximum of two evacuations (incidence of 3.7%), and it was promptly resolved conservatively using ultrasound-guided needle aspiration, without the need for reoperation. The other minor complication was a case of necrosis recorded in a patient with a skin-reducing mastectomy pattern (3.7% incidence in the totality of cases) that was cared for conservatively by debridement and wound packing with rapid resolution.

At a mean follow-up of 7.3 months (range 3–11 months), among major complications, we recorded only one case of implant infection that required implant removal. In this patient, the prosthesis was explanted and followed by multiple washes, which, together with systemic antibiotic treatment, allowed preserving the skin flap. No occurrence of dehiscence or hematoma was recorded, as well as capsular contractures (Table 2).

**Table 2:** Complications divided into minor complications.

Complications	Major	Minor
Seroma	0 (0)	1 (3.7)
Dehiscence	0 (0)	0 (0)
Hematoma	0 (0)	0 (0)
Necrosis	0 (0)	1 (3.7)
Infection	1 (3.7)	0 (0)
Capsular contracture	0 (0)	0 (0)
Total	1 (3.7)	2 (7.4)

**Figure 2:**
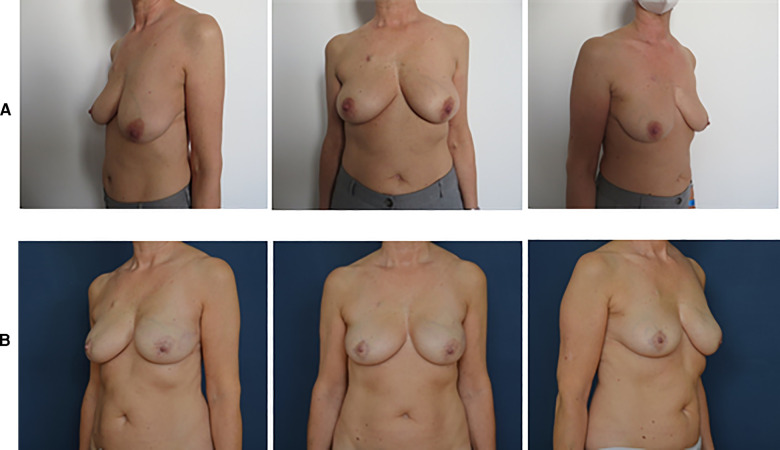
Pre- and postoperative pictures of BRAXON^®^*Fast*-assisted PPBRs. (**A**) Patient with left breast cancer: preoperative pictures. (**B**) Patient with left breast cancer: 8 months after left prepectoral reconstruction surgery with BRAXON^®^*Fast* and breast implant, contralateral reduction mammoplasty.

**Figure 3:**
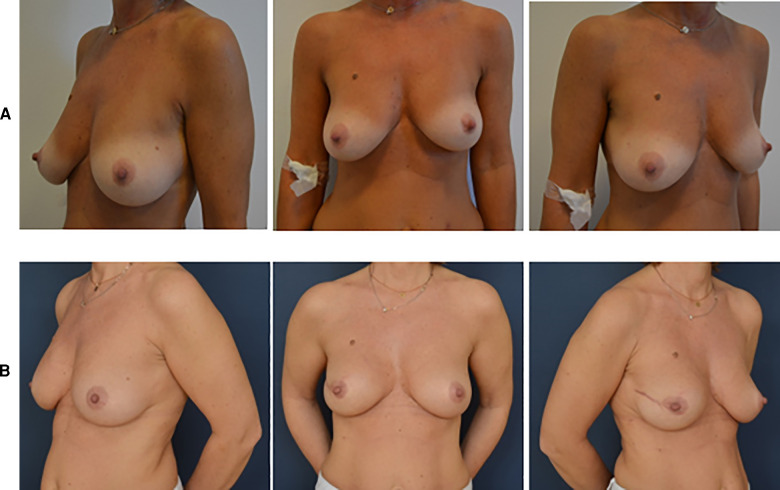
Pre- and postoperative pictures of BRAXON^®^*Fast*-assisted PPBRs. (**A**) Patient with right breast cancer: preoperative pictures. (**B**) Patient with left breast cancer: 10 months after right prepectoral reconstruction surgery with BRAXON^®^*Fast* and breast implant.

Natural aesthetic results are reported in Figures 2, 3.

## Discussion

Development of new production technologies in the biomaterial field led to the release, in 2021, of a novel version of BRAXON® ADM: BRAXON®*Fast* (licensed by DECOMED, Venezia, Italy), which is the first three-dimensional matrix indicated for PPBR. It is a preshaped collagen membrane with a shell-like conformation, already curved to follow the convexities typical of the anterior surface of a breast implant.

Twenty-three patients underwent one-stage PPBR with BRAXON®*Fast* 3D-ADM at “Ca’ Foncello” Hospital of Treviso, Italy. The overall minor complication rate was 7.4%, and only one major complication occurred in the form of an infection, accounting for 3.7% of all cases.

Until recently, most breast reconstructions were performed in two stages—with the placement of a tissue expander and then an implant—or in one stage but below the pectoralis major muscle, causing patients to have more postoperative pain, discomfort, and animation deformity (18). In PPBR, the pectoral muscle lift is avoided, and as reported by Maruccia *et al.*, patients experience less pain, a faster return to normal activity, and better QoL than patients who have undergone subpectoral reconstruction (11). In breast reconstruction, dermis-derived acellular matrices constitute a layer between the skin and the silicone implant, augmenting soft tissue thickness and providing a regenerative scaffold, unlike synthetic meshes that solely constitute a mechanical support and cause fibrotic reactions (19).

The choice of the most apt biological material in breast reconstruction appears to be crucial in determining complications and results. In our case series, complication rates can be considered moderate-low, in particular seroma, which accounts for 3.7%. In fact, a recent experience with a biological matrix is that of Hansson and colleagues, who used a two-stage technique obtaining a 38% rate of seroma formation with the bovine pericardium, 10 times higher compared to our outcomes with a thin, natural, porcine ADM (20). Moreover, early experiences with suboptimally processed cadaveric ADMs and xenogeneic matrices with added chemicals showed that such products could irritate tissues and cause an increased exudate production (5, 21). Indeed, although an older review reports a higher rate of seroma formation with ADM compared to a completely submuscular expander, an up-to-date review by Caputo *et al.* finds no increment in seroma formation with the use of BRAXON® and proposes operative strategies to improve outcomes further (17, 21). Similarly, more recent data on PPBR performed with human ADMs on 2,270 breasts confirmed the low rate of seroma formation (4.8%) (22). Additionally, the rate of mastectomy flap necrosis recorded in our case series is in line with the result of such meta-analysis (3.7% and 3.4%, respectively). In contrast, the latter reports high rates of infection (7.9%) compared to our data (3.7%) (22).

In 2013, Fisher and colleagues reviewed a large series of 14,585 patients who had received breast reconstruction and found that bilateral operations were associated with a higher risk of implant loss. In our case series, implant loss occurred in one reconstruction only due to a unilateral implant infection occurring in a bilateral patient. Although the samples are in no way comparable between this collection and Fisher's findings, they do appear in accordance (23). In the same publication, the authors found a significant difference in surgery duration between cases with and without implant failure. Although in multivariate analysis, time did not surface as an ascertained risk factor, it is still an important variable for a safe outcome. In our experience, we did not record a difference in the overall surgery duration with the use of BRAXON® or BRAXON®*Fast*, but the exposure time of the ADM and of the implant was reduced to about one-third. In fact, if the classic BRAXON® matrix required 25–30 stitches for the ADM preparation (for a total of 15 min), only 7–10 stitches are needed (for a total of 6 min) now with BRAXON®*Fast*.

BRAXON®*Fast* innovation consists of ready-to-use volume and projection. The lens-shape conformation of the anterior surface, as a dome, easily adapts to the breast implant silhouette without the need for tailoring. The unfenestrated posterior flap of the ADM provides the total biological interface between the implant and the pectoralis major muscle. The prosthesis is rapidly and easily inserted into the BRAXON®*Fast* shell, and by suturing the two ADM flaps together, the prosthesis is completely hidden from the patient's tissues. BRAXON®*Fast* is available in multiple sizes, designed to accommodate various implant volumes: our cohort of patients required prostheses ranging from 180 cc to 570 cc; therefore, all three BRAXON®*Fast* sizes were used. According to our experience, BRAXON®*Fast* speeds up the implant wrapping process without multiple intricate and time-consuming wire-passing operations characterizing other devices that could put sterility at stake.

BRAXON®*Fast* is then meant to be fixed both at the pectoralis fascia and at the subcutaneous tissue to obviate malposition and rotation of the implant. Moreover, the experience of Mura *et al.* reports that fixing the matrix to the subcutaneous tissue can be an effective way to obliterate spaces and prevent the accumulation of inflammation-derived fluids resulting from the surgical insult of mastectomy (24). In our center, this practice has been applied since the very early cases with BRAXON® total implant coverage, and in this case series, the small number of seroma occurrences (one breast) counts for the 3.7% of all treated breasts. Although this is a limited court, this practice appears effective in controlling dead-spaces obliteration and implant fixation.

The introduction of the acellular dermal matrix in 2005 by Breuing and Warren as an adjunct in subpectoral implant-based reconstructions has seen a large increase in its use for IBR following mastectomies (3, 25). Since then, the application of ADM has found a different purpose and technique with the total implant coverage started by the muscle-sparing reconstruction technique of Berna and others published in 2014 (5). This same prepectoral reconstruction with the Braxon technique has been extensively studied in the last decade, with a plethora of publications defining patient selection criteria and proving it safe and effective with BRAXON® ADM (26, 27).

This article reports on using new-generation three-dimensional collagen membranes in the current prepectoral breast surgery practice. Reduced overall exposure time and implant preparation were the main practical implications. Overall, the outcomes are in line with those reported by the iBAG study, the world's biggest data collection on PPBR (1,450 cases) performed with BRAXON® ADM (26).

Nevertheless, this is a single-center retrospective analysis from a prospectively maintained database of a small patients’ cohort and will suffer the disadvantages of a retrospective study. We also did not evaluate patient's satisfaction. A longer follow-up is needed to assess long-term complications. Further investigation is granted.

Our series represents an early experience with BRAXON®*Fast* in IBR with a medium-short follow-up. The device demonstrated an excellent safety profile, and the outcomes show an effective performance combining very good clinical aspects and optimal aesthetic results.

## Data Availability

The raw data supporting the conclusions of this article will be made available by the authors without undue reservation.
